# Assessing knowledge and attitudes toward childhood obesity among Syrian refugees in Al Za’atari refugee camp: a quantitative approach

**DOI:** 10.3389/fpubh.2026.1793282

**Published:** 2026-04-02

**Authors:** Balqees Shahin, Nour Mahrouseh, Nasha’at Al-Afeer, Orsolya Varga

**Affiliations:** 1 Department of Public Health and Epidemiology, Faculty of Medicine, University of Debrecen, Debrecen, Hungary; 2 Independent Data Analyst, Amman, Jordan

**Keywords:** Al Za’atari camp, BMI-for-age z-score, childhood obesity, food insecurity, parental knowledge and attitudes, Syrian refugees

## Abstract

**Background:**

Childhood obesity is a growing global concern, particularly among conflict-affected populations. In contexts such as Al Za’atari Refugee Camp, monotonous diets, dependence on food aid, and low levels of physical activity all increase the risk of obesity. When combined with low health literacy, these conditions contribute to a double burden of malnutrition. Yet little is known about how displaced families themselves understand childhood obesity.

**Objective:**

This study aimed to evaluate the knowledge, attitudes and practice of Syrian refugee parents and their children (aged 6–18 years) concerning childhood obesity in the Al Za’atari Refugee Camp.

**Methods:**

A cross-sectional quantitative survey was conducted among 381 Syrian refugee households (*N* = 762; 381 parents and 381 children). A structured questionnaire was used to collect data on demographic characteristics, knowledge, attitudes, and practices related to childhood obesity. The questionnaire was administered using the KOBO Toolbox system. Statistical analyses included descriptive statistics and multivariable linear regression and mediation analyses.

**Results:**

Overall, 34.1% of children were overweight and 30.5% were obese. Higher parental nutritional knowledge was associated with lower child BMI-for-age Z-scores (BAZ) (*β* = −2.104; 95% CI: −3.641 to −0.567) and healthier dietary behavior (*β* = −0.761; 95% CI: −0.848 to −0.673). Boys and older children had lower BAZ than girls and younger peers. Household food insecurity showed a weak, non-significant positive association with BAZ. Higher parental BMI was significantly related to higher child BAZ, whereas self-reported family history of obesity was not. Parental attitudes and feeding practices were both associated with children’s dietary behavior; feeding practices showed only small, non-significant mediation of the attitude’s behavior link. Socioeconomic instability especially parental unemployment and reliance on informal or unstable income was strongly associated with higher BAZ and poorer dietary behavior, while more stable employment was linked to healthier outcomes.

**Conclusion:**

In this refugee camp setting, childhood obesity is highly prevalent and closely linked to parental nutritional knowledge, parental BMI, and household economic conditions, more than to food insecurity alone. Family-focused, gender-responsive interventions that combine caregiver nutrition education with economic stability and access to healthy foods are critical to reducing obesity risk among displaced children.

## 1 Introduction

Childhood obesity has emerged as one of the most pressing global public health challenges, contributing significantly to the growing burden of noncommunicable diseases such as type 2 diabetes, cardiovascular conditions, and poor psychosocial outcomes (1). Importantly, children with obesity are more likely to remain obese into adulthood, which carries an increased risk of lifelong morbidity and premature mortality (2, 3). Despite international calls for prevention, prevalence rates continue to rise worldwide, with the steepest increases observed in low- and middle-income countries and in fragile, conflict-affected settings (4, 5).

Refugee and displaced populations are uniquely vulnerable to both undernutrition and obesity, reflecting the “double burden of malnutrition” (6). Studies have shown that food insecurity and constrained access to fresh, diverse foods often drive reliance on inexpensive, energy-dense diets, thereby increasing obesity risk even when caloric intake appears sufficient (7, 8). Refugee children in particular face additional constraints such as overcrowding, limited safe spaces for physical activity, and dependency on humanitarian food assistance, which collectively exacerbate obesogenic environments (9, 10).

Parental influences represent another critical determinant of children’s nutritional and behavioral outcomes. Evidence consistently shows that parents’ knowledge, attitudes, and practices (KAP) shape dietary behaviors, feeding practices, and children’s activity levels (11, 12). Misperceptions of child weight status and cultural norms that normalize excess body weight are particularly common in Middle Eastern contexts and may undermine prevention efforts (13, 14). Furthermore, household structure and socioeconomic instability have been identified as strong predictors of childhood obesity risk, often outweighing short-term behavioral factors (15, 16). Evidence indicates that children in food-insecure households face not only immediate risks such as micronutrient deficiencies, illness, and stunted growth, but also adverse cognitive, behavioral, and economic outcomes later in life (9, 17). These risks persist into adolescence, where unhealthy eating patterns such as low fruit and vegetable intake, frequent snacking, and high consumption of sugary beverages contribute to rising obesity prevalence (5, 9).

Given the rapid growth of displaced populations with an estimated 117.3 million people forcibly displaced globally by mid-2025 (18), and close to 50 million children displaced due to conflict and violence by the end of 2024 (19), and the limited research in humanitarian contexts, there is an urgent need to better understand the complex interplay of parental knowledge, household food insecurity, socioeconomic constraints, and children’s behaviors in shaping obesity outcomes. This study addresses these gaps by examining multiple determinants of childhood obesity among Syrian refugee children living in Al Za’atari Camp, Jordan. Specifically, it investigates: (1) the association between parental nutritional knowledge and children’s dietary behaviors and nutritional status; (2) the relationship between household food insecurity and obesity-promoting dietary patterns; (3) the alignment or mismatch between parental attitudes and practices; (4) the role of parental BMI and family history of obesity in explaining variation in children’s BMI-for-age Z-scores (BAZ); and (5) the association of socioeconomic and household factors to obesity risk in this refugee setting.

## 2 Methodology

### 2.1 Study design and sample

A cross-sectional survey was conducted between 15 April 2025 and 15 July 2025 among Syrian refugee families residing in Al Za’atari Refugee Camp, Jordan. The camp is one of the largest refugee settlements globally, hosting an estimated 51,163 (20) residents and approximately 13,500 households. The camp is characterized by limited access to diverse foods, recreational facilities, and healthcare services.

The study population consisted of parents and their children aged 6–18 years living in the camp. Eligibility required that households included at least one child within this age group and that both the parent and child were willing to participate. Households without children in the specified age range or those unwilling to provide consent were excluded. Eligibility was confirmed using screening questions at the start of the questionnaire, where parents self-reported the presence of at least one child aged 6–18 years in the household and confirmed their role as the parent or legal guardian. Only respondents meeting these criteria were able to proceed with the survey.

Given the contextual constraints of conducting research in a refugee setting, a convenience sampling strategy was employed. Recruitment was facilitated by trained Syrian refugee volunteers who distributed the survey link through established WhatsApp and Facebook community networks.

The sample size was calculated using the single-proportion formula with finite population correction (21), and verified in OpenEpi (version 3.01) (22). Assuming a 95% confidence level (*Z* = 1.96), 5% margin of error (*e* = 0.05), and an expected proportion of 50% (*p* = 0.50) in a finite population of 13,500 households, the required sample size was 374. Allowing for a 7% non-response rate, the target sample was approximately 402 households. We obtained 381 complete parent–child dyads, which, while slightly below the inflated target, exceeded the minimum required sample size and was adequate for the planned regression analyses (23, 24).

### 2.2 Data collection

Data was collected using a structured, anonymous questionnaire administered through the KOBO Toolbox platform, enabling secure, real-time data entry. The questionnaire was developed by adapting and integrating items from validated instruments (9, 12, 25). Additional constructs were informed by the USDA Food Security Core Module (26), and a nutrition knowledge tool originally developed by Parmenter and Wardle (27).

The survey was self-administered online, with responses entered directly by participants, no identifying information was collected. Trained field volunteers were available in the camp to provide technical and procedural support (e.g., clarifying instructions and assisting with access to the platform) when needed, without influencing participants’ responses. The questionnaire was developed in English, translated into Arabic, and back translated to ensure linguistic and conceptual equivalence. To maintain data quality, households were instructed to complete the questionnaire only once, the survey platform restricted multiple submissions from the same device.

## 3 Measures

The instrument assessed key determinants of childhood obesity; see Appendix 1 for the full questionnaire and sources. It included questions on demographic and household characteristics, covering parental age, sex, education, employment status, household size and structure, as well as income sources and the main income earner. Parental and child anthropometric information including self-reported height and weight were also collected and later used to calculate parental BMI and children’s BAZ in accordance with WHO growth standards (28).

Another section focused on parental nutritional knowledge, with items assessing the ability to interpret nutrition labels, identifying nutrient-rich foods, and recognizing healthy dietary patterns. Higher scores indicated greater knowledge. This was complemented by items exploring parental attitudes toward child weight and obesity, including cultural beliefs and weight-related perceptions. These questions included reverse-coded statements such as *“as long as a child is happy, it does not matter if the child is overweight,”* allowing for the assessment of both favorable and unfavorable attitudes.

In addition, the questionnaire addressed parental practices by capturing nutrition-related parenting practices such as food control, regulation of snacking, encouragement of healthy eating, and promotion of children’s physical activity. Higher practice scores reflected more obesity-preventive orientations. To assess the child environment more directly, the tool also measured children’s health and behaviors, including fruit and vegetable intake, consumption of sugary drinks and milk, frequency of snacking, and meal skipping, as well as screen time and physical activity participation. Responses were later combined into composite indices, with higher values representing healthier practices.

Finally, household food insecurity was assessed through adapted USDA items, which examined the affordability of balanced meals, the frequency of skipped meals, and whether children or adults in the household experienced hunger due to limited resources.

Although the questionnaire was administered online, measures were taken to ensure clarity and inclusiveness across diverse households. These included simplifying item wording, pre-testing the questionnaire to ensure comprehension, and applying rigorous translation and back-translation procedures.

### 3.1 Data analysis

All analyses were conducted using IBM SPSS Statistics (version 26), Stata (version 15), and R for anthropometric calculations based on the WHO 2007 growth reference (AnthroPlus algorithm) (28). The dataset was screened prior to analysis for duplicate entries, implausible anthropometric values, and incomplete responses. Questionnaires with >20% missing data were excluded from the analytic sample. Continuous variables were assessed for normality, and categorical variables were harmonized across domains to ensure consistent coding.

Descriptive statistics were generated to characterize the sample in terms of parental demographics, socioeconomic indicators, household food insecurity, parental nutrition knowledge, and children’s dietary and lifestyle behaviors. Means and standard deviations were reported for continuous variables, and frequencies and percentages for categorical variables. Children’s BAZ were computed using the WHO 2007 growth reference (28). Children were classified as normal weight (≤ + 1 SD), overweight (> + 1 to ≤ + 2 SD), or obese (> + 2 SD). Parental BMI was calculated from self-reported height and weight.

To capture key behavioral constructs, composite indices were created. A Child Obesogenic Behavior Score was derived by aggregating items on snacking frequency, sugary drink consumption, meal skipping, and screen time. Items were standardized and averaged to yield scores ranging from 0 to 1, with higher scores indicating more obesogenic behaviors. A Household Food Insecurity Score was computed from adapted USDA items, with higher scores indicating greater food insecurity.

Multivariable regression models were used to address the study aims. Linear regression examined associations between parental nutrition knowledge and children’s behaviors and BAZ; between household food insecurity and children’s behaviors and BAZ; and between parental BMI/family obesity history and child BAZ. Mediation analysis was conducted to evaluate whether parental practices mediated associations between parental attitudes and children’s dietary and activity behaviors, using the average causal mediation effect framework. Socioeconomic and household characteristics (e.g., employment status, household type, and primary income source) were also modeled as predictors of children’s behaviors and BAZ.

All models were adjusted for child age and sex. A two-tailed significance threshold of *p* < 0.05 was applied, and 95% confidence intervals were reported alongside effect estimates.

### 3.2 Ethical consideration

The research project received approval from the Regional and Institutional Research Ethics Committee (No. 7120A-2025). Participation was voluntary and the questionnaire was completed anonymously; no identifying information was collected. Because the survey was anonymous and minimal risk, consent was obtained electronically from parents/guardians prior to participation. Completion and submission of the questionnaire indicated consent. Children completed the child section under parental permission; participation was voluntary and participants could discontinue at any time.

## 4 Results

### 4.1 Sample characteristics and descriptive results

A total of 381 households were included in the final analysis (Table 1). The initial sample consisted of 407 respondents, of whom 26 were excluded in the data processing phase due to incomplete data, including missing child age or unreported responses. The final sample comprised 381 parents aged 21–72 years (mean age 42.52 ± 9.59 years) and 381 children aged 6–18 years (mean age 13.78 ± 2.56 years). Among parents, 53.0% were male and 47.0% were female, while 46.0% of children were male and 54.0% were female.

**Table 1:** Sociodemographic, economic, and behavioral characteristics of the study sample (*n* = 381 households).

Variable	Category	*n* (mean ± SD)	%
Parent age (years)	Mean ± SD	42.52 ± 9.59	–
Child age (years)	Mean ± SD	13.78 ± 2.56	–
Parent gender	Male	201 (44.12 ± 10.33)	53.0
	Female	180 (40.73 ± 8.37)	47.0
Child gender	Male	174 (13.72 ± 2.65)	46.0
	Female	207 (13.84 ± 2.48)	54.0
Type of household	Couple family with children alone or with additional household members	330	86.61
	Female or male single parent with children (no additional members)	46	12.07
	Female single parent with or without children with additional household members	5	1.31
Family history of obesity	Yes	177	46.5
	No	204	53.5
Employment status of main earner	Employed (full-time or part-time)	235	61.7
	Unemployed/seeking work	125	32.8
	Other/not in labor force	21	5.4
Main income source	Aid organizations	79	20.73
	Self-employment and/or wages and working with aid organizations	228	59.84
	Self-employment and/or wages and aid from aid organizations	74	19.42
Food security status	Food secure	60 (−1.87 ± 0.51)	15.7
	Mild food insecurity	78 (−0.46 ± 0.31)	20.5
	Moderate food insecurity	201 (0.45 ± 0.18)	52.8
	Severe food insecurity	42 (1.38 ± 0.29)	11.0
Child BMI-for-age Z-score category	Normal weight (≥ − 2 SD to ≤ + 1 SD)	133 (−0.01 ± 0.74)	34.9
	Overweight (> + 1 SD to ≤ + 2 SD)	130 (1.53 ± 0.29)	34.1
	Obese (> + 2 SD)	116 (2.79 ± 1.43)	30.5
	Underweight (≥ − 3 SD to < − 2 SD)	1 (−2.50)	0.3
	Severe thinness (< −3 SD)	1 (−4.46)	0.3
Child behavioral score	Healthy behavior (≥ 67th percentile)	112 (0.73 ± 0.05)	29.4
	Moderate behavior (33rd–66th percentile)	255 (0.51 ± 0.08)	66.9
	Unhealthy behavior (≤ 33rd percentile)	14 (0.29 ± 0.05)	3.7
Parent–child dyad composition and their mean knowledge percentage	Mother–daughter	103	40
	Mother–son	77	71
	Father–daughter	104	71
	Father–son	97	54

Food insecurity was categorized using Z-scores: Z > +1 (severe), 0 to +1 (moderate), −1 to 0 (mild), and Z < −1 (food secure). Health behaviors were classified by percentile distribution as healthy (≥67th percentile), moderate (33rd–66th percentile), and unhealthy (≤33rd percentile). Parental weight status was defined using BMI (kg/m^2^) as underweight (<18.5), normal weight (18.5–24.9), overweight (25.0–29.9), and obesity (≥30.0). Child nutritional status was classified using BMI-for-age Z-scores (BAZ) as severe thinness (<−3 SD), underweight (−3 to <−2 SD), normal weight (−2 to +1 SD), overweight (> + 1 to +2 SD), and obese (> + 2 SD).

Most households were couple families with children with or without additional household members (86.61%). Female or male single-parent households with children accounted for 12.07% of the sample, whereas females single-parent households with or without children but with additional household members were uncommon 1.31%. A family history of obesity was reported by 46.5% of respondents, while 53.5% reported no such history.

Regarding economic characteristics, 61.7% of households reported an employed main income earner (full-time or part-time), whereas 32.8% were unemployed or seeking work, and 5.4% were classified as not in the labor force. The most frequently reported main income source was self-employment and/or wage-based income combined with working with aid organizations, 59.84%, while 20.73% reported no independent income, and receiving aid from organizations.

In terms of food security, the mean score for the food security index was 17.22 (SD 3.60) with a minimum score of 4 and a maximum of 25, where higher scores indicate greater food insecurity. The mean value corresponds approximately to the moderate food insecurity range. Because this food security index does not have fixed cutoff points, z-scores were used to classify households into food security levels. Households were categorized using standardized Z-scores of the food security index (see Table 1 legend). Within this context, 15.7% of households were classified as food secure, 20.5% experienced mild food insecurity, 52.8% reported moderate food insecurity, and 11.0% experienced severe food insecurity. In parallel, based on BAZ with a mean of 1.350 (1.50 SD), 34.9% of children were classified as normal weight, 34.1% as overweight, and 30.5% as obese. Notably underweight and severe thinness was rare; each observed in 0.3% of children.

Beyond nutritional status, assessment of child health-related behaviors showed a mean of 0.570 (0.135 SD) with minimum 0.182 score and maximum score of 0.864. 29.4% of children were classified as having healthy behaviors, 66.9% had moderate behaviors, and 3.7% were classified as having unhealthy behaviors.

Finally, with respect to parent–child dyad composition, the sample included 103 mother–daughter dyads with a mean parent–child knowledge score of 40%, 77 mother–son dyads with a mean knowledge score of 71%, 104 father–daughter dyads with a mean knowledge score of 71%, and 97 father–son dyads with a mean knowledge score of 54% (total *n* = 381 dyads). The overall mean parent–child knowledge score across all dyads was 58%, representing the weighted average knowledge score for the full sample.

### 4.2 Parental nutritional knowledge and children’s dietary behavior and nutritional status

Linear regression models were fitted to examine associations between parental nutrition knowledge and (i) children’s BAZ scores and (ii) children’s obesogenic behavior score, adjusting for child age and sex. Higher parental nutrition knowledge was significantly associated with lower child BAZ (*β* = −2.104, *p* = 0.007; 95% CI: −3.641 to −0.567). Male children had lower BAZ than females (*β* = −0.407, *p* = 0.016; 95% CI: −0.738 to −0.075), and older age was also inversely associated with BAZ (*β* = −0.025, *p* = 0.004; 95% CI: −0.042 to −0.008).

In a separate model, higher parental nutrition knowledge was strongly associated with a lower (less obesogenic) child behavior score (*β* = −0.761, *p* < 0.001; 95% CI: −0.848 to −0.673), indicating fewer unhealthy behaviors such as meal skipping and frequent snacking. Child sex was not significantly associated with the behavior score (*β* = 0.017, *p* = 0.077; 95% CI: −0.005 to 0.040).

### 4.3 Household food insecurity and obesity-promoting behaviors

The association between household food insecurity and children’s nutritional and behavioral outcomes was examined while controlling for child age and sex. Household food insecurity was positive but not significantly associated with children’s BAZ.

Food insecurity showed a weak and non-significant relationship with children’s dietary behavior. Children in more food-insecure households demonstrated slightly poorer dietary behavior (*β* = −0.002, *p* = 0.240), though the association was not statistically significant. Male children exhibited marginally lower behavioral scores (*β* = −0.028, *p* = 0.053), while older children tended to report slightly healthier behaviors (*β* = 0.001, *p* = 0.089).

### 4.4 Parental attitudes, feeding practices, and child behaviors

A mediation analysis was conducted to examine whether parental feeding practices mediated the relationship between parents’ attitudes toward childhood obesity and their children’s dietary behaviors. Parental attitudes were operationalized as beliefs minimizing concern about childhood weight (e.g., “*a happy child does not need to worry about weight*”), while feeding practices reflected parental control and encouragement of healthy eating and activity habits. The regression model examining the effect of parental attitudes on feeding practices was not statistically significant. Parents’ general attitudes toward child weight did not significantly predict their reported feeding practices (*β* = 0.05, *p* = 0.375).

The second regression model, which included both parental attitudes and feeding practices as predictors of child behavior, was highly significant. Both variables were negatively associated with child dietary behavior, indicating that more permissive attitudes and less structured feeding practices were linked to unhealthier eating patterns. Specifically, parental attitudes (*β* = −0.024, *p* = 0.001) and feeding practices (*β* = −0.063, *p* < 0.001) were each inversely related to children’s healthy behavior scores.

The mediation analysis revealed that feeding practices partially mediated the relationship between parental attitudes and child behavior, although the indirect effect was small and not statistically significant (ACME = −0.003, 95% CI: −0.011 to 0.004). The direct effect of parental attitudes on child behavior remained significant (*β* = −0.024, 95% CI: −0.037 to −0.010), accounting for most of the total effects (Total Effect = −0.027, 95% CI: −0.043 to −0.011). Approximately 13% (95% CI: 8–32%) of the total effect of parental attitudes on child behavior was mediated through feeding practices.

### 4.5 Parental BMI, family history of obesity, and child nutritional status

To examine whether parental weight status or a family history of obesity was associated with children’s BAZ, two separate regression analyses were performed. Higher parental BMI predicted higher child BAZ (*β* = 0.030, *p* = 0.033; 95% CI: 0.003, 0.058), indicating that children of parents with higher body mass indices tended to have greater BAZ.

Family history of obesity, reported as the presence of obesity among first-degree relatives, was not significantly associated with children’s BAZ. The coefficient was small and non-significant (*β* = 0.025, *p* = 0.874; 95% CI: −0.281, 0.331), suggesting no link between reported familial obesity history and children’s current BAZ.

### 4.6 Socioeconomic and household factors associated with childhood obesity

To examine how parental socioeconomic characteristics and household composition relate to children’s nutritional status and dietary behavior, two regression analyses were conducted; that included the assessment of household type, current labor status, and income source to assess their association with BAZ or children dietary behavior.

The association between socioeconomic and household variables with BAZ showed significant association with unemployed parents in their labor status (*β* = 0.706, *p* < 0.001; 95% CI: 0.384, 1.028) compared to employed parents. Income source from aid organizations (*β* = −0.443, *p* = 0.025; 95% CI: −0.828, −0.057) compared to self-employment and/or wages and working with aid organizations.

Socioeconomic and household variables were also significantly associated with children’s dietary behavior. Household type as being part of female or male single parent with children has significant negative association with child dietary behavior index and belonging to a household where female is a single parent with/without children and with additional household members (*β* = −0.094, *p* = <0.001; 95% CI: −0.125, −0.062 and *β* = −0.074, *p* = 0.007; 95% CI: −0.127, −0.021, respectively) compared to household with couple family with children alone or with additional members. Employment status emerged as a key determinant. Children of unemployed parents demonstrated an association with dietary behavior (*β* = 0.047, *p* = 0.002; 95% CI: 0.017, 0.077), compared to those whose parents were employed.

Income source also played a role: reliance solely on aid was linked with less healthy behaviors (*β* = −0.075, *p* < 0.001; 95% CI: −0.117, −0.034), while mixed income from self-employment and/or wages and aid from organizations was similarly associated with lower dietary quality (*β* = −0.097, *p* < 0.001; 95% CI: −0.124, −0.071) (Table 2).

**Table 2:** Multivariate linear regression and mediation analyses examining predictors of BMI-for-age Z-score and dietary behavior scores among refugee children (*n* = 381).

Outcome	Model/Research question	Predictor	*β* (Coefficient)	*p*-value	95% CI
BMI-for-age Z-score	Parental nutritional knowledge and child BMI-for-age Z-scores (BAZ)	Parental knowledge score	−2.104	0.007	(−3.641, −0.567)
		Child gender (Male)	−0.407	0.016	(−0.738, −0.075)
		Child age	−0.025	0.004	(−0.042, −0.008)
Dietary Behavior Score	Parental nutritional knowledge and child dietary behavior	Parental knowledge score	−0.761	<0.001	(−0.848, −0.673)
		Child gender (Male)	0.017	0.077	(−0.005, 0.040)
		Child age	0.001	0.148	(−0.0003, 0.002)
BMI-for-age Z-score	Household food insecurity and obesity-promoting behaviors	Food insecurity score	0.028	0.122	(−0.008, 0.064)
		Child gender (Male)	−0.508	<0.001	(−0.792, −0.224)
		Child age	−0.025	0.005	(−0.042, −0.008)
Dietary Behavior Score	Household food insecurity and child dietary behavior	Food insecurity score	−0.002	0.240	(−0.007, 0.002)
		Child age	0.001	0.089	(−0.0002, 0.003)
		Child gender (Male)	−0.028	0.053	(−0.056, 0.000)
Dietary Behavior Score	Parental attitudes, feeding practices, and child behaviors (Mediation analysis)	Parental attitudes	−0.024	0.001	(−0.037, −0.010)
		Feeding practices	−0.063	<0.001	(−0.075, −0.052)
		Average Causal Mediation Effect	−0.003	–	(−0.011, 0.004)
		Direct Effect	−0.024	–	(−0.037, −0.010)
		Total Effect	−0.027	–	(−0.043, −0.011)
		% of Total Effect Mediated	13%	–	(8–32%)
BMI-for-age Z-score	Parental BMI	Parental BMI	0.030	0.033	(0.003, 0.058)
		Child gender (Male)	−0.432	0.005	(−0.733, −0.130)
		Child age	−0.027	0.002	(−0.044, −0.010)
	family obesity history	Family history of obesity	0.025	0.874	(−0.281, 0.331)
BMI-for-age Z-score	Socioeconomic and household factors	Household type: Female or male single parent with children (reference: Couple family with children alone or with additional household members)	−0.300	0.340	(−0.919, 0.318)
		Household type: Female single parent with/without children and with additional household members (reference: Couple family with children alone or with additional household members)	−0.602	0.123	(−1.369, 0.165)
		Labor status: Unemployed parent (reference: Employed parent)	0.706	<0.001	(0.384, 1.028)
		Labor status: Others (reference: Employed parent)	−0.209	0.456	(−0.761, 0.342)
		Income source: Aid organizations (reference: Self-employment and/or wages and working with aid organizations)	−0.443	0.025	(−0.828, −0.057)
		Income source: Self-employment and/or wages and aid from aid organizations (reference: Self-employment and/or wages and working with aid organizations)	−0.196	0.299	(−0.568, 0.175)
Dietary Behavior Score	Socioeconomic and household factors	Household type: Female or male single parent with children (reference: Couple family with children alone or with additional household members)	−0.094	<0.001	(−0. 125, −0.062)
		Household type: Female single parent with/without children and with additional household members (reference: Couple family with children alone or with additional household members)	−0.074	0.007	(−0. 127, −0.021)
		Labor status: Unemployed parent (reference: Employed parent)	0.047	0.002	(0.017, 0.077)
		Labor status: Others (reference: Employed parent)	−0.036	0.172	(−0.088, 0.016)
		Income source: Aid organizations (reference: Self-employment and/or wages and working with aid organizations)	−0.075	<0.001	(−0.117, −0.034)
		Income source: Self-employment and/or wages and aid from aid organizations (reference: Self-employment and/or wages and working with aid organizations)	−0.097	<0.001	(−0.124, −0.071)

This table presents the results of multiple linear regression models examining the associations between parental nutritional knowledge, household food insecurity, parental attitudes and feeding practices, parental BMI, and socioeconomic characteristics with child BMI-for-age Z-score (BAZ) and dietary behavior score. *β* coefficients are reported together with 95% confidence intervals (CI) and p-values. Positive *β* coefficients indicate a direct association between the predictor and the outcome, whereas negative coefficients indicate an inverse association. Reference categories were Female (child gender), unemployed parent (employment status), and self-employment/wages plus aid (income source). Household food insecurity and parental BMI were analyzed as continuous variables. Family history of obesity was coded as Yes = 1 and No = 0 (reference). A significance level of *p* < 0.05 was applied.

## 5 Discussion

This study examined the determinants of childhood obesity among Syrian refugee children living in Al Za’atari Camp, focusing on behavioral, familial, and socioeconomic influences within a standardized humanitarian food environment. Childhood overweight and obesity were highly prevalent, exceeding estimates reported among Syrian refugees in Lebanon and Turkey, indicating that the camp has progressed along the humanitarian nutrition-transition continuum where caloric sufficiency coexists with poor dietary quality (29, 30).

Gender and age were significant correlates of child nutritional status. Boys had significantly lower BAZ than girls (*β* = −0.407, *p* = 0.016), while older age was inversely associated with BAZ (*β* = −0.025, *p* = 0.004). These differences are likely driven by sociocultural and environmental factors rather than biological mechanisms. In many Arab contexts, boys have greater access to outdoor spaces and organized physical activities, including NGO-supported sports programs, whereas girls often face cultural norms and safety constraints that limit mobility and physical activity (31). Regional evidence consistently documents higher physical inactivity among girls due to conservative gender norms and inadequate built environments (31). Older children, in contrast, may exercise greater autonomy in dietary and activity choices, potentially moderating parental influence on weight outcomes. These associations are also plausible within refugee-camp environments such as Al Za’atari, where high population density, limited safe play spaces, and safety concerns may restrict opportunities for physical activity, particularly among girls. Similar environmental and social barriers to physical activity have been documented among refugee children living in constrained settlement environments and low-resource settings (32, 33).

Parental nutritional knowledge, parental BMI, and socioeconomic instability emerged as the strongest determinants of children BAZ scores and dietary behavior, while household food insecurity and parental attitudes played a less central, largely indirect role. The findings illustrate the layered influence of individual, household, and structural factors shaping malnutrition in protracted displacement settings, where undernutrition and excess weight can coexist (34).

Higher parental nutritional knowledge was strongly associated with lower child BAZ and healthier dietary behaviors, even after adjusting for child age and gender. Specifically, each unit increase in parental knowledge was linked to a reduction in child BAZ (*β* = −2.104, *p* = 0.007) and improvements in dietary behavior scores (*β* = −0.761, *p* < 0.001). These findings align with the Behavior Change Wheel framework, which conceptualizes behavior as arising from interactions between capability, opportunity, and motivation (35). In this context, parental nutritional knowledge enhances psychological capability and motivation, enabling healthier food choices despite constrained opportunities related to food availability and economic hardship. Within refugee-camp environments, where households often depend partly on standardized food assistance and low-cost foods available through camp markets, nutritional knowledge may help caregivers make more effective use of available foods and reduce reliance on energy-dense, nutrient-poor options (36, 37).

A school-based nutrition intervention among Syrian refugee children in Lebanon demonstrated improvements in dietary knowledge and eating patterns, suggesting that informed caregivers and children can better optimize limited resources (9). Similarly, studies among displaced populations show that higher nutritional literacy is associated with improved dietary diversity and reduced reliance on calorie-dense staple foods even under financial constraints (38). These findings highlight the potential of education-focused interventions to partially offset structural limitations through improved decision-making, portion control, and food prioritization.

Parental BMI was a strong predictor of child BAZ, highlighting intergenerational transmission of obesity risk. Each unit increase in parental BMI was associated with higher child BAZ, reflecting shared dietary practices, activity patterns, and stress-related behaviors within households. These findings are consistent with extensive literature demonstrating that parental adiposity captures both genetic susceptibility and shared environmental exposures (39). Although self-reported family history of obesity was not statistically significant in this study, similar refugee-based research suggests that shared routines and constrained food environments may outweigh hereditary factors in shaping child weight outcomes (40).

Socioeconomic instability emerged as one of the strongest determinants of child obesity risk. Children from unemployed or aid-dependent households exhibited higher BAZ and poorer dietary behaviors, while those from part-time or wage-earning households demonstrated healthier outcomes. Economic precarity limits purchasing power, disrupts meal planning, and promotes reliance on low-cost, energy-dense foods mechanisms well established in nutritional economics (8, 41). These pathways align with the regional nutrition transition, characterized by increased consumption of ultra-processed foods and reduced access to safe physical activity spaces (34, 42).

Although household type was not a statistically significant predictor in regression models, extended and multigenerational living arrangements may still influence feeding practices in camp settings. International evidence indicates that grandparents and extended caregivers often use food as an expression of care during hardship, fostering permissive feeding environments (43, 44). Similar associations between adolescent obesity and parental employment conditions have been reported in Jordan (45), reinforcing the role of economic stability rather than household size alone.

Household food insecurity was positively but not significantly associated with child BAZ (*β* = 0.028, *p* = 0.122) and showed a weak, non-significant association with dietary behavior (*β* = −0.002, *p* = 0.240). Although statistically non-significant, the direction of these associations is consistent with the hunger–obesity paradox, whereby households experiencing chronic food insecurity rely on inexpensive, energy-dense, nutrient-poor foods (34, 46). This phenomenon reflects the broader double burden of malnutrition, in which undernutrition and obesity coexist within the same populations.

Overall, these findings should also be interpreted within the broader framework of the double burden of malnutrition, in which undernutrition and excess weight coexist within the same populations and sometimes within the same households. In protracted refugee settings such as Al Za’atari Camp, improvements in caloric availability through food assistance programs may occur alongside persistent limitations in dietary diversity and micronutrient intake, creating conditions where overweight and obesity emerge without eliminating risks of nutrient deficiencies. This pattern reflects a nutritional transition typical of humanitarian environments where constrained food choices and reliance on food assistance shape both undernutrition and obesity risks simultaneously (34, 36). From a programmatic perspective, addressing childhood obesity in refugee camps requires both education-based and structural interventions. Nutrition education delivered through schools and primary health clinics may help caregivers optimize available foods, while structural approaches such as improving access to affordable nutrient-dense foods and expanding safe opportunities for physical activity may be necessary to address environmental constraints (37).

Within Al Za’atari Camp, the lack of statistical significance likely reflects homogeneous deprivation across households, all of which face restricted food choice and heavy reliance on standardized aid distributions. When scarcity is nearly universal, variation in dietary quality rather than caloric quantity becomes the dominant driver of obesity risk. Similar patterns have been observed in European populations, where food insecurity did not independently predict childhood obesity after socioeconomic factors were controlled (47).

### 5.1 Implications for intervention

Taken together, these findings indicate that effective obesity prevention in refugee camps must extend beyond caloric adequacy. Interventions should adopt family-focused and gender-responsive approaches that combine caregiver nutrition education with strategies to enhance household economic stability and access to healthier foods. Integrating nutrition education with livelihood support, cash-transfer programs, and culturally appropriate physical-activity opportunities may help bridge the gap between knowledge and practice. Such multi-component strategies are essential for addressing childhood obesity within the structural constraints of humanitarian settings and for promoting long-term nutritional resilience among displaced populations.

### 5.2 Strengths and limitations

This study provides one of the few quantitative assessments of childhood obesity determinants in a protracted refugee camp setting, integrating multiple behavioral, familial, and socioeconomic dimensions. A key strength lies in its use of validated instruments to measure parental nutritional knowledge, attitudes, and feeding practices, as well as food insecurity allowing for nuanced interpretation of behavioral and structural interactions.

Nevertheless, several limitations warrant consideration. First, the study’s cross-sectional design limits causal inference, as associations between parental and child factors cannot establish temporal or directional effects. Second, self-reported measures of parental knowledge, attitudes, and practices may be subject to recall or social desirability bias, potentially leading to underreporting of obesogenic behaviors. Third, children’s height and weight were self-reported, which may introduce measurement error and could affect BMI and BAZ classification as well as estimates of malnutrition prevalence. However, in Al-Zaatari refugee camp, families commonly maintain official records that include children’s anthropometric measurements for school enrollment and health services, and children are regularly measured at local health clinics within each district; therefore, many reported values were likely based on recent documented measurements rather than estimation. Nevertheless, some degree of reporting error cannot be excluded.

Fourth, the dataset was confined to one refugee camp, which may limit external generalizability to other displaced or host-community populations with different food systems and socio-political contexts. While convenience sampling may limit the generalizability of findings, it provided a practical and culturally appropriate means of engagement within the camp context. Additionally, dietary and physical-activity data relied on brief behavioral indicators rather than objective measurements (e.g., accelerometry or 24-h dietary recall), constraining the precision of lifestyle assessment. Also, recruitment relied primarily on WhatsApp and Facebook, which may have introduced selection bias by favoring households with reliable digital access and familiarity with social media platforms, potentially underrepresenting families with limited connectivity or digital literacy.

Finally, while the analytical framework adjusted for major confounders such as age, gender, and parental BMI, unmeasured factors including psychosocial stress, maternal education, or access to healthcare, may also mediate obesity risk. Despite these limitations, the study offers a comprehensive, evidence-based perspective that advances understanding of the complex interplay between structural deprivation, family dynamics, and childhood obesity within humanitarian settings.

## Data Availability

The raw data supporting the conclusions of this article will be made available by the authors, without undue reservation.
