# Supplementary material for: Assessing knowledge and attitudes toward childhood obesity among Syrian refugees in Al Za’atari refugee camp: a quantitative approach
Source: Front Public Health. 2026 Apr 2;14:1793282. doi: 10.3389/fpubh.2026.1793282 (PMC13083096; doi:10.3389/fpubh.2026.1793282)
Supplement: Supplementary file 1 [file Data_Sheet_1.pdf]

Child Obesity Questionnaire

Welcome and thank you for taking the time to participate in this survey. This questionnaire aims to explore the knowledge and perceptions of childhood obesity among parents and their children living in the Al Zaatari refugee camp. Please note that your responses are completely anonymous, and by completing this survey, you give your consent for the data provided to be used for research purposes for a PhD project to study the knowledge of Syrian refugees on childhood obesity. We appreciate your valuable contribution to this important study, for more information please contact Balqeessha96@gmail.com.

Parents

» Section 1: Personal Information

Gender *

- ○ Female
- ○ Male

Age *

Height *

Weight *

» Section 2: Household Structure

Type of Household *

- ◯ Couple family with children
- ◯ Couple family without children
- ◯ Couple family with children and additional people
- ◯ Couple family without children but with additional people
- ◯ Female single parent with children (no additional people)
- ◯ Female single parent without children (with additional people)
- ◯ Female single parent with children (with additional people)
- ◯ Male single parent with children (no additional people)
- ◯ Male single parent without children (with additional people)
- ◯ Male single parent with children (with additional people)
- ◯ Single with additional people
- ◯ Single without additional people

Family history of (Obesity) *

- ◯ Yes
- ◯ No

» KAP Questions

» » Knowledge (K)

Do you read the nutrition labels on food products? *

◯ Strongly Disagree — ◯ Disagree — ◯ Agree — ◯ Strongly Agree

Do you find it difficult to understand the details printed on nutrition labels? *

◯ Strongly Disagree — ◯ Disagree — ◯ Agree — ◯ Strongly Agree

Do you have good knowledge of what constitutes a healthy and nutritious diet for children? *

Strongly Disagree | Disagree | Agree | Strongly Agree

» » » Attitude (A)

Do you believe that as long as a child is happy, it does not matter if the child is overweight? *

Strongly Disagree | Disagree | Agree | Strongly Agree

Do you think being overweight as a child has no implications on the child's health as an adult? *

Strongly Disagree | Disagree | Agree | Strongly Agree

Do you agree that obesity is only a health concern for adults? *

Strongly Disagree | Disagree | Agree | Strongly Agree

» » » »Parctices (P)

Do you actively control what your children consume? *

Strongly Disagree | Disagree | Agree | Strongly Agree

Do you encourage your children to spend time on physical activities every day? *

Strongly Disagree | Disagree | Agree | Strongly Agree

Do you encourage your children to join sports or extracurricular physical activities at school? *

Strongly Disagree | Disagree | Agree | Strongly Agree

» Family Eating Habits

Do all individuals in the household eat at the same table, not necessarily at the same time? *

- Yes
- No
- Not sure
- Refuse to answer

» Employment Status Questionnaire

» » Section 1: Household Income

How many individuals in the household earn an income through employment? *

Who is the main income earner in the family? *

- Mother
- Father
- Child (Male)
- Child (Female)
- Children (Combined)

» » » Section 2: Employment Status

What is the current employment status of the main income earner? *

- Full-time
- Part-time
- Unemployed/Looking for work
- Student
- Retired
- Other (Specify):

Other *

What is the current employment status of the second income earner? (If applicable, select one):

- ○ Full-time
- ○ Part-time
- ○ Unemployed/Looking for work
- ○ Student
- ○ Retired
- ○ Other (Specify):

other *

» » » »Section 3: Family Employment Details

How many members of your family have a job and earn income? *

Your Occupation: *

Main Income Earner's Occupation *

From which of the following sources did your household receive income in the past 12 months? (Select all that apply): *

- ☐ Wages and Salaries
- ☐ Income from Self-Employment
- ☐ Money from Aid Organizations

» Nutrition Knowledge Questionnaire (Parga, 1999)

Which of the following are components of a healthy diet? (Select all that apply): *

- [ ] Bread and cereals
- [ ] Fats and oils
- [ ] Fruits and vegetables
- [ ] Poultry, meat, and fish

Which of the following is a good source of calcium? *

- ○ Bread
- ○ Cheese
- ○ Chicken
- ○ Citrus fruit

What is the recommended percentage of total fat intake in a diet? *

- ○ 10%
- ○ 30%
- ○ 45%
- ○ 50%

Which of the following is the best source of protein? *

- ○ Pasta
- ○ Potatoes
- ○ Rice and beans
- ○ Poultry, meat, and fish

Vitamins and minerals are classified as: *

- ○ Supplements
- ○ Calories
- ○ Extra energy
- ○ Micronutrients

Which of the following is high in carbohydrates? *

- Fish
- Steak
- Rice and black beans
- Salad dressing (Ranch)

RDAs are recommendations for nutrient requirements for: *

- Most healthy people
- Only sick people
- Telling you the amount of vitamins and minerals you need to achieve
- Advising you on how to eat when on a diet

Which of the following contains the highest number of calories per gram? *

- 1 gram of alcohol
- 1 gram of carbohydrate (CHO)
- 1 gram of fat
- 1 gram of protein

Which of the following is the best source of iron? *

- Citrus fruits
- Turkey
- Scrambled eggs
- Yogurt

Which of the following helps prevent heart disease? *

- PUFA (Polyunsaturated fatty acids)
- Sodium/Salt
- Saturated fat
- Unsaturated fat

Which of the following is a source of Vitamin D? *

- Liver
- Mangoes
- Oranges
- Cereal (cornflakes)

Which of the following is considered a mineral? *

- Riboflavin
- Vitamin C
- Calcium
- Thiamin

Which of the following is a good source of Vitamin B12? *

- Black beans
- Meat
- Spinach
- Pears

Which of the following has the highest fat content? *

- 3 ounces of beef
- 3 ounces of chicken
- 3 ounces of cheddar cheese
- 3 ounces of creamy salad dressing

Which type of fat is most beneficial for preventing heart disease? *

- PUFA (Polyunsaturated Fatty Acids)
- Saturated fat
- Unsaturated fat

Which of the following is true about fiber? *

- Fiber is needed to provide energy
- Fiber is not necessary in the diet
- Fiber helps lower cholesterol in the body
- Fiber helps the body regulate temperature

Which of the following is the best source of fiber? *

- ◯ Baked potato with margarine
- ◯ Spaghetti and meat sauce
- ◯ Chicken and yellow rice
- ◯ Rice and black beans

Which of the following is a source of Vitamin D? *

- ◯ Mangoes
- ◯ Oranges
- ◯ Cereal (cornflakes)

Which of the following is considered a mineral? *

- ◯ Riboflavin
- ◯ Vitamin C
- ◯ Calcium
- ◯ Thiamin

Adolescents need: *

- ◯ More calories, vitamins, and minerals than adults
- ◯ Fewer calories, vitamins, and minerals than adults
- ◯ Only more vitamins than adults
- ◯ The same nutritional needs as adults

Which of the following is a good source of Vitamin B12? *

- ◯ Black beans
- ◯ Meat
- ◯ Spinach
- ◯ Pears

Folic acid is: *

- ◯ A vitamin necessary to prevent defects in the fetus during pregnancy
- ◯ A food additive that helps keep food fresh
- ◯ A waste product of metabolism

What is an antioxidant? *

- ◯ A substance needed by the body to kill germs
- ◯ A chemical used to make food fat-free
- ◯ A chemical needed by people over 50
- ◯ A substance needed by the body to prevent damage to cells

How many servings of fruits and vegetables should one have daily? *

- ◯ At least 1 serving of each
- ◯ 2 to 3 servings of each
- ◯ 3 servings of fruits and 1 serving of vegetables
- ◯ 4 to 5 servings of fruits and 2 servings of vegetables

A calorie is a fatty substance found in food that causes weight gain. *

- ◯ TRUE
- ◯ FALSE

Margarine contains fewer calories than butter. *

- ◯ TRUE
- ◯ FALSE

High intakes of certain vitamins can be very harmful. *

- ◯ TRUE
- ◯ FALSE

Alcohol contains no calories because it is not a food. *

- ◯ TRUE
- ◯ FALSE

The fat in foods is what makes you fat. *

- ◯ TRUE
- ◯ FALSE

How often did this happen? *

- Never
- Sometimes true
- Often true
- I don't know/Refuse to answer

How often did this happen? *

- Never
- Sometimes true
- Often true
- I don't know/Refuse to answer

How often did this happen? *

- Never
- Sometimes true
- Often true
- I don't know/Refuse to answer

Did your child/children not eat enough because you couldn't afford enough food? *

- Never
- Sometimes true
- Often true
- I don't know/Refuse to answer

How often did adults in the household cut or skip meals due to lack of food? *

- Almost every month (3+ months)
- Some months (2 or fewer months)
- I don't know/Refuse to answer

Did you personally eat less than you felt you should because there wasn't enough money for food? *

- Yes
- No
- I don't know/Refuse to answer

Were you ever hungry but did not eat because you couldn't afford enough food? *

- ◯ Yes
- ◯ No
- ◯ I don't know/Refuse to answer

Did you lose weight because there wasn't enough money for food? *

- ◯ Yes
- ◯ No
- ◯ I don't know/Refuse to answer

Did you or other adults in your household not eat for a whole day because there wasn't enough money for food? *

- ◯ Yes (More than 3 months)
- ◯ No (Less than 3 months)
- ◯ I don't know/Refuse to answer

Did you ever cut the size of your child's meals due to lack of money? *

- ◯ Yes (More than 3 months)
- ◯ No (Less than 3 months)
- ◯ I don't know/Refuse to answer

Did any of the children in your household skip meals because there wasn't enough money for food? *

- ◯ Yes (More than 3 months)
- ◯ No (Less than 3 months)
- ◯ I don't know/Refuse to answer

Was your child ever hungry because you couldn't afford enough food? *

- ◯ Yes (More than 3 months)
- ◯ No (Less than 3 months)
- ◯ I don't know/Refuse to answer

Did your child ever not eat for a whole day because there wasn't enough money for food? *

- ◯ Yes (More than 3 months)
- ◯ No (Less than 3 months)
- ◯ I don't know/Refuse to answer

For Their Children

» Personal Information

Age *

Height *

Weight *

-

- ◯ -
- ◯ -

» » Dietary and lifestyle Knowledge attitude and behavior (KAB) evaluation items

» » » Knowledge (0-15)

Fruits and Vegetables are part of a healthy diet. *

- Yes
- No

It is important to eat different kinds of fruits every week. *

- Yes
- No

Eating breakfast is an important part of a healthy lifestyle. *

- Yes
- No

Is snacking important between meals? *

- Yes
- No

An example of a healthy snack is _? *

- Fruits
- Chocolate
- Candies
- Chips
- Soft drinks

From which type of food should you eat least? *

- Bread, rice and pasta
- Milk, cheese and yogurt
- Sweets, fats and oils
- Fruits and vegetables
- Meat , chicken and eggs
- I don't know

How many servings of fruits and vegetables should you have per day? *

- One
- 2-3
- 4-5
- 5 or more
- I don't know

Eating breakfast helps me do well at school. *

- Yes always
- Sometimes
- Never

The best fluid for my body is : *

- Water
- Sweetened juice
- Soft drinks
- All of the above
- I don't know
- Yes

Sweetened bottled/canned juice and soft drinks cause dental caries. *

- No
- I don't know
- Yes

Chocolate and candies cause dental caries. *

- No
- I don't know
- Yes

Eating a lot of sweets makes me gain weight. *

- No
- I don't know
- It keeps you healthy

Being physically active is important because *

- It gives you energy
- It makes you happy
- Improves your academic performance
- All of the above

» » » Attitudes (0-10)

I think healthy food tastes good *

Agree Not Sure Disagree

I think eating healthy is very important *

Agree Not Sure Disagree

I believe my health in future may be affected by what I eat today *

Agree Not Sure Disagree

I think eating breakfast every day is good for my health *

Agree Not Sure Disagree

Drinking a glass of milk everyday is good for my health *

Agree Not Sure Disagree

I think drinking a glass of water every day is good for my health *

Agree — Not Sure — Disagree

I think Fruits and vegetables are good for my health *

Agree — Not Sure — Disagree

I think water is the best fluid for my body *

Agree — Not Sure — Disagree

I think chips are very high in salt *

Agree — Not Sure — Disagree

I think soft drinks are very high in sugar *

Agree — Not Sure — Disagree

» » » Practices (0-22)

How often do you eat vegetables? *

- Several times a day
- Once a day
- 2-3 times/week
- Once a week
- Never

How often do you eat fruits? *

- Several times a day
- Once a day
- 2-3 times/week
- Once a week
- Never

How often did you skip meals? *

- Several times a day
- Once a day
- 2-3 times/week
- Once a week
- Never

How often did you drink milk or eat milk products such as Labneh, cheese or yogurt? *

- Several times a day
- Once a day
- 2-3 times/week
- Once a week
- Never

Over the past month, how often do you watch TV at home? *

- ○ Everyday
- ○ 4-6 days/week
- ○ 2-3 days/week
- ○ 1 day/week
- ○ Never

Are you able to snack between meals? *

- ○ Yes
- ○ No

Are you able to consume 3 meals per day (breakfast, lunch and dinner) ? *

- ○ Yes
- ○ No

استبيان المعرفة عن السمنة في مرحلة الطفولة

بالترحيب بكم وشكرًا على تخصيص وقتكم للمشاركة في هذا الاستبيان. يهدف هذا الاستبيان إلى استكشاف المعرفة والمفاهيم المتعلقة بالسمنة لدى الأطفال بين الأهالي وأطفالهم المقيمين في مخيم الزعتري للاجئين.
يرجى ملاحظة أن إجاباتكم ستبقى مجهولة تمامًا، ومن خلال إكمال هذا الاستبيان فإنكم تعطون موافقتكم لاستخدام البيانات المقدمة لأغراض بحثية ضمن مشروع دكتوراه يهدف إلى دراسة معرفة اللاجئين السوريين حول السمنة لدى الأطفال.
نُقدّر مساهمتكم القيّمة في هذا البحث الهام، ولمزيد من المعلومات يرجى التواصل عبر البريد الإلكتروني التالي: Balqeessha96@gmail.com.

الأهالي

» القسم 1: المعلومات الشخصية

جنس رب الأسرة *

- ◯ أنثى
- ◯ ذكر

العمر *

الطول *

الوزن *

» القسم الثاني: هيكل الأسرة

نوع الأسرة *

- أسرة زوجين مع أطفال
- أسرة زوجين بدون أطفال
- أسرة زوجين مع أطفال وأشخاص إضافيين
- أسرة زوجين بدون أطفال ولكن مع أشخاص إضافيين
- أم عزباء مع أطفال (دون أشخاص إضافيين)
- أم عزباء بدون أطفال (مع أشخاص إضافيين)
- أم عزباء مع أطفال (مع أشخاص إضافيين)
- أب عازب مع أطفال (دون أشخاص إضافيين)
- أب عازب بدون أطفال (مع أشخاص إضافيين)
- أب عازب مع أطفال (مع أشخاص إضافيين)
- عازب مع أشخاص إضافيين
- عازب دون أشخاص إضافيين

وجود تاريخ عائلي للإصابة بالسمنة *

- نعم
- لا

» أسئلة المعرفة والمواقف والممارسات (KAP)

» » المعرفة (K):

هل تقرأ الملصقات الغذائية على المنتجات؟ *

أختلف بشدة — أختلف — أوافق — أوافق بشدة

هل تجد صعوبة في فهم التفاصيل المطبوعة على الملصقات الغذائية؟ *

أختلف بشدة — أختلف — أوافق — أوافق بشدة

* هل لديك معرفة جيدة بما يشكّل نظامًا غذائيًا صحيًا ومغذيًا للأطفال؟

أختلف بشدة — أختلف — أوافق — أوافق بشدة

» » » المواقف (A):

* هل تعتقد أنه طالما أن الطفل سعيد، فلا بأس إذا كان يعاني من زيادة في الوزن؟

أختلف بشدة — أختلف — أوافق — أوافق بشدة

* هل تعتقد أن زيادة الوزن في مرحلة الطفولة لا تؤثر على صحة الطفل في المستقبل؟

أختلف بشدة — أختلف — أوافق — أوافق بشدة

* هل توافق على أن السمنة تُعدّ مصدر قلق صحي فقط للبالغين؟

أختلف بشدة — أختلف — أوافق — أوافق بشدة

» » »الممارسات (P):

* هل تتحكم بشكل فعّال في ما يستهلكه أطفالك من طعام؟

أختلف بشدة — أختلف — أوافق — أوافق بشدة

* هل تشجّع أطفالك على ممارسة الأنشطة البدنية يوميًا؟

أختلف بشدة — أختلف — أوافق — أوافق بشدة

* هل تحثّ أطفالك على المشاركة في الرياضات أو الأنشطة البدنية اللامنهجية في المدرسة؟

أختلف بشدة — أختلف — أوافق — أوافق بشدة

» عادات الأسرة الغذائية

*
هل يتناول جميع أفراد الأسرة طعامهم على نفس الطاولة، حتى وإن لم يكن في نفس الوقت؟

- نعم
- لا
- لست متأكدًا
- أرفض الإجابة

» استبيان الحالة الوظيفية

» » القسم الأول: دخل الأسرة

*
كم عدد الأفراد في الأسرة الذين يحصلون على دخل من العمل؟

*
من هو المعيل الرئيسي في الأسرة؟

- أم
- أب
- طفل (ذكر)
- طفل (أنثى)
- الأطفال (مجموع)

» » القسم الثاني: الحالة الوظيفية

*
ما هي الحالة الوظيفية الحالية للمعيل الرئيسي؟

- دوام كامل
- دوام جزئي
- عاطل عن العمل/باحث عن عمل
- طالب
- متقاعد
- أخرى (حدد):

*
أخرى

ما هي الحالة الوظيفية الحالية للمعيل الثاني؟ (إن وُجد، يُرجى اختيار واحدة):

- دوام كامل
- دوام جزئي
- عاطل عن العمل/باحث عن عمل
- طالب
- متقاعد
- أخرى (حدد):

*

أخرى

» » »القسم الثالث: تفاصيل العمل لدى الأسرة

*

كم عدد أفراد أسرتك الذين لديهم وظيفة ويتقاضون دخلًا؟

*

مهنة المعيل الرئيسي:

*

الدخل من العمل الرئيسي

*

من أي المصادر التالية حصلت أسرتك على دخل في الـ 12 شهرًا الماضية؟ (اختر كل ما ينطبق):

- الأجور والرواتب
- الدخل من العمل الحر
- المال من المنظمات الإنسانية

» استبيان معرفة التغذية (بارغا، 1999)

*
أي من العناصر التالية هي مكونات لنظام غذائي صحي؟ (اختر كل ما ينطبق):

- ☐ الخبز والحبوب
- ☐ الدهون والزيوت
- ☐ الفواكه والخضروات
- ☐ الدواجن، اللحوم، والأسماك

*
أي من العناصر التالية يعتبر مصدرًا جيدًا للكالسيوم؟

- ○ الخبز
- ○ الجبن
- ○ الدجاج
- ○ الحمضيات

*
ما هي النسبة الموصى بها من الدهون الإجمالية في النظام الغذائي؟

- ○ 0.1
- ○ 0.3
- ○ 0.45
- ○ 0.5

*
أي من العناصر التالية هو أفضل مصدر للبروتين؟

- ○ المعكرونة
- ○ البطاطا
- ○ الأرز والفاصوليا
- ○ الدواجن واللحوم والأسماك

*
تصنف الفيتامينات والمعادن على أنها:

- ○ المكملات
- ○ السعرات الحرارية
- ○ الطاقة الزائدة
- ○ المغذيات الدقيقة

أي من العناصر التالية يحتوي على نسبة عالية من الكربوهيدرات؟ *

- السمك
- الستيك
- الأرز والفاصوليا السوداء
- صلصة السلطة (رانش)

التوصيات اليومية (RDA) هي توصيات لاحتياجات العناصر الغذائية لـ: *

- أكثر الأشخاص صحة
- فقط الأشخاص المرضى
- إخبارك بكمية الفيتامينات والمعادن التي تحتاج لتحقيقها
- نصيحتك بشأن كيفية الأكل أثناء اتباع الحمية

أي من العناصر التالية يحتوي على أعلى عدد من السعرات الحرارية لكل جرام؟ *

- 1 جرام من الكحول
- 1 جرام من الكربوهيدرات (CHO)
- 1 جرام من الدهون
- 1 جرام من البروتين

أي من العناصر التالية هو أفضل مصدر للحديد؟ *

- الفواكه الحمضية
- الدجاج التركي
- بيض مخفوق
- لبن زبادي

أي من العناصر التالية يساعد في الوقاية من أمراض القلب؟ *

- PUFA (الأحماض الدهنية المتعددة غير المشبعة)
- الصوديوم/الملح
- الدهون المشبعة
- الدهون غير المشبعة

*
أي من العناصر التالية هو مصدر لفيتامين د؟

- الكبد
- المانجو
- البرتقال
- حبوب الإفطار (رقائق الذرة)

*
أي من العناصر التالية يعتبر معدنًا؟

- الريبوفلافين
- فيتامين C
- الكالسيوم
- الثيامين

*
أي من العناصر التالية هو مصدر جيد لفيتامين B12؟

- الفاصوليا السوداء
- اللحم
- السبانخ
- الكمثرى

*
أي من العناصر التالية يحتوي على أعلى محتوى من الدهون؟

- 3 أونصات من اللحم البقري
- 3 أونصات من الدجاج
- 3 أونصات من جبن الشيدر
- 3 أونصات من صلصة السلطة الكريمية

*
أي نوع من الدهون هو الأكثر فائدة للوقاية من أمراض القلب؟

- PUFA (الأحماض الدهنية المتعددة غير المشبعة)
- الدهون المشبعة
- الدهون غير المشبعة

*
أي من العناصر التالية صحيح بشأن الألياف؟

- الألياف ضرورية لتوفير الطاقة
- الألياف غير ضرورية في النظام الغذائي
- الألياف تساعد في خفض الكوليسترول في الجسم
- الألياف تساعد الجسم في تنظيم درجة الحرارة

أي من العناصر التالية هو أفضل مصدر للألياف؟ *

- بطاطا مشوية مع السمن
- سباغيتي مع صلصة اللحم
- دجاج مع أرز أصفر
- أرز وفاصوليا سوداء

أي من العناصر التالية هو مصدر لفيتامين د؟ *

- مانجو
- برتقال
- حبوب الإفطار (رقائق الذرة)

أي من العناصر التالية يعتبر معدنًا؟ *

- ريبوفلافين
- فيتامين C
- كالسيوم
- ثيامين

يحتاج المراهقون إلى: *

- مزيد من السعرات الحرارية، الفيتامينات، والمعادن أكثر من البالغين
- أقل من السعرات الحرارية، الفيتامينات، والمعادن عن البالغين
- فقط المزيد من الفيتامينات عن البالغين
- نفس احتياجات التغذية مثل البالغين

أي من العناصر التالية هو مصدر جيد لفيتامين B12؟ *

- الفاصوليا السوداء
- اللحم
- السبانخ
- الكمثرى

حمض الفوليك هو: *

- فيتامين ضروري لمنع التشوهات في الجنين أثناء الحمل
- إضافة غذائية تساعد في الحفاظ على الطعام طازجاً
- منتج نفايات من الأيض

ما هو مضاد الأكسدة؟ *

- مادة يحتاجها الجسم لقتل الجراثيم
- مادة كيميائية تُستخدم لجعل الطعام خالٍ من الدهون
- مادة كيميائية يحتاجها الأشخاص فوق ٥٠ سنة
- مادة يحتاجها الجسم لمنع تلف الخلايا

كم عدد الحصص من الفواكه والخضروات يجب أن يتناولها الشخص يوميًا؟ *

- على الأقل حصة واحدة من كل نوع
- ٢ إلى ٣ حصص من كل نوع
- ٣ حصص من الفواكه وحصة واحدة من الخضروات
- ٤ إلى ٥ حصص من الفواكه و٢ حصص من الخضروات

السعرات الحرارية هي مادة دهنية توجد في الطعام وتسبب زيادة الوزن. *

- صحيح
- خطأ

المارغرين يحتوي على سعرات حرارية أقل من الزبدة. *

- صحيح
- خطأ

الاستهلاك المفرط لبعض الفيتامينات قد يكون ضارًا جدًا. *

- صحيح
- خطأ

الكحول لا يحتوي على سعرات حرارية لأنه ليس طعامًا. *

- صحيح
- خطأ

الدهون في الطعام هي ما يجعلك سمينًا. *

- صحيح
- خطأ

*
الطعام الذي تم شراؤه لم يدم طويلاً

- أبداً
- أحيانًا صحيح
- غالبًا صحيح
- لا أعرف / أرفض الإجابة

*
لم أستطع تحمل تكلفة الوجبات المتوازنة

- أبدًا
- أحيانًا صحيح
- غالبًا صحيح
- لا أعرف / أرفض الإجابة

*
لم أستطع إطعام الأطفال وجبات متوازنة

- أبدًا
- أحيانًا صحيح
- غالبًا صحيح
- لا أعرف / أرفض الإجابة

*
هل لم يأكل طفلك/أطفالك بما فيه الكفاية بسبب عدم قدرتك على تحمل تكلفة الطعام؟

- أبدًا
- أحيانًا صحيح
- غالبًا صحيح
- لا أعرف / أرفض الإجابة

*
كم مرة قام البالغون في الأسرة بتقليص أو تخطي الوجبات بسبب نقص الطعام؟

- تقريبًا كل شهر (أكثر من 3 أشهر)
- بعض الأشهر (شهرين أو أقل)
- لا أعرف / أرفض الإجابة

*
هل تناولت أنت شخصيًا طعامًا أقل مما تشعر أنه يجب أن تأكله بسبب عدم توفر المال للطعام؟

- نعم
- لا
- لا أعرف / أرفض الإجابة

هل كنت جائعًا في أي وقت ولكنك لم تأكل لأنك لم تتمكن من تحمل تكلفة الطعام؟ *

- نعم
- لا
- لا أعرف / أرفض الإجابة

هل فقدت الوزن بسبب عدم توفر المال للطعام؟ *

- نعم
- لا
- لا أعرف / أرفض الإجابة

هل أنت أو أي شخص بالغ آخر في أسرتك لم يتناول الطعام طوال اليوم بسبب عدم توفر المال للطعام؟ *

- نعم (أكثر من 3 أشهر)
- لا (أقل من 3 أشهر)
- لا أعرف / أرفض الإجابة

هل قمت بتقليص حجم وجبات طفلك بسبب نقص المال؟ *

- نعم (أكثر من 3 أشهر)
- لا (أقل من 3 أشهر)
- لا أعرف / أرفض الإجابة

هل تخطى أي من الأطفال في أسرتك الوجبات بسبب نقص المال للطعام؟ *

- نعم (أكثر من 3 أشهر)
- لا (أقل من 3 أشهر)
- لا أعرف / أرفض الإجابة

هل كان طفلك جائعًا في أي وقت لأنك لم تتمكن من تحمل تكلفة الطعام؟ *

- نعم (أكثر من 3 أشهر)
- لا (أقل من 3 أشهر)
- لا أعرف / أرفض الإجابة

هل لم يأكل طفلك طوال اليوم بسبب عدم توفر المال للطعام؟ *

- نعم (أكثر من 3 أشهر)
- لا (أقل من 3 أشهر)
- لا أعرف / أرفض الإجابة

لأطفالهم

» المعلومات الشخصية

العمر *

الطول *

الوزن *

جنس الطفل

- ذكر
- أنثى

» » تقييم المعرفة والمواقف والسلوك (KAB) الغذائي ونمط الحياة

» » » المعرفة (من 0 إلى 15)

الفواكه والخضروات جزء من النظام الغذائي الصحي. *

- نعم
- لا

من المهم تناول أنواع مختلفة من الفواكه كل أسبوع. *

- نعم
- لا

تناول الإفطار جزء مهم من نمط الحياة الصحي. *

- نعم
- لا

هل الوجبات الخفيفة مهمة بين الوجبات؟ *

- نعم
- لا

*
مثال على وجبة خفيفة صحية هو _؟

- فواكه
- شوكولاتة
- حلويات
- رقائق بطاطس
- مشروبات غازية

*
من أي نوع من الطعام يجب أن تأكل أقل؟

- خبز، أرز ومعكرونة
- حليب، جبن ولبن
- حلويات، دهون وزيوت
- فواكه وخضروات
- لحم، دجاج وبيض
- لا أعرف

*
كم عدد حصص الفواكه والخضروات التي يجب أن تتناولها يوميًا؟

- واحدة
- 2-3
- 4-5
- 5 أو أكثر
- لا أعرف

*
تناول الإفطار يساعدني على التفوق في المدرسة.

- دائمًا نعم
- أحيانًا
- أبدًا

*
أفضل سائل لجسمي هو:

- ماء
- عصير محلى
- مشروبات غازية
- جميع ما سبق
- لا أعرف
- نعم

*
العصائر المعلبة/المعبأة المحلاة والمشروبات الغازية تسبب تسوس الأسنان.

- لا
- لا أعرف
- نعم

*
الشوكولاتة والحلويات تسبب تسوس الأسنان.

- لا
- لا أعرف
- نعم

*
تناول الكثير من الحلويات يجعلني أكتسب الوزن.

- لا
- لا أعرف
- نعم

*
النشاط البدني مهم لأنه:

- يمنحك الطاقة
- يجعلك سعيدًا
- يحسن أدائك الأكاديمي
- جميع ما ذكر

» » » المواقف (من 0 إلى 10)

*
أعتقد أن الطعام الصحي طعمه جيد

موافق — غير متأكد — غير موافق

*
أعتقد أن تناول الطعام الصحي مهم جدًا

موافق — غير متأكد — غير موافق

*
أعتقد أن صحتي في المستقبل قد تتأثر بما أتناوله اليوم

موافق — غير متأكد — غير موافق

*
أعتقد أن تناول الإفطار كل يوم جيد لصحي

موافق | غير متأكد | غير موافق

*
شرب كوب من الحليب كل يوم جيد لصحي

موافق | غير متأكد | غير موافق

*
أعتقد أن شرب كوب من الماء كل يوم جيد لصحي

موافق | غير متأكد | غير موافق

*
أعتقد أن الفواكه والخضروات جيدة لصحي

موافق | غير متأكد | غير موافق

*
أعتقد أن الماء هو أفضل سائل لجسمي

موافق | غير متأكد | غير موافق

*
أعتقد أن الشيبس يحتوي على نسبة عالية من الملح

موافق | غير متأكد | غير موافق

*
أعتقد أن المشروبات الغازية تحتوي على نسبة عالية من السكر

موافق | غير متأكد | غير موافق

» » » الممارسات (من 0 إلى 22)

كم مرة تأكل الخضروات؟ *

- عدة مرات في اليوم
- مرة في اليوم
- ٢-٣ مرات في الأسبوع
- مرة في الأسبوع
- أبدًا

كم مرة تأكل الفواكه؟ *

- عدة مرات في اليوم
- مرة في اليوم
- ٢-٣ مرات في الأسبوع
- مرة في الأسبوع
- أبدًا

كم مرة تخطيت الوجبات؟ *

- عدة مرات في اليوم
- مرة في اليوم
- ٢-٣ مرات في الأسبوع
- مرة في الأسبوع
- أبدًا

كم مرة شربت الحليب أو تناولت منتجات الحليب مثل اللبنة أو الجبن أو الزبادي؟ *

- عدة مرات في اليوم
- مرة في اليوم
- ٢-٣ مرات في الأسبوع
- مرة في الأسبوع
- أبدًا

في الشهر الماضي، كم مرة كنت تشاهد التلفاز في المنزل؟ *

- كل يوم
- ٤-٦ أيام في الأسبوع
- ٢-٣ أيام في الأسبوع
- يوم في الأسبوع
- أبدًا

هل تستطيع تناول وجبات خفيفة بين الوجبات؟ *

- نعم
- لا

هل تستطيع تناول 3 وجبات يوميًا (الإفطار، الغداء والعشاء)؟ *

- نعم
- لا
